# Evaluating Patient Satisfaction and Quality of Life After Undergoing Laparoscopic Cholecystectomy in Al-Qunfudhah Governorate, Saudi Arabia

**DOI:** 10.7759/cureus.62276

**Published:** 2024-06-12

**Authors:** Medhat Taha, Bader Ali Aleesa, Muteb Ali Algarni, Saeed Mohammed Alomari, Salem Hussain Alzubidy

**Affiliations:** 1 Department of Anatomy, Umm Al-Qura University, Al-Qunfudhah, SAU; 2 College of Medicine and Surgery, Umm Al-Qura University, Al-Qunfudhah, SAU

**Keywords:** saudi arabia, complications, patients perception, quality of life, satisfaction, laparoscopic cholecystectomy

## Abstract

Background: Gallstone disease, commonly referred to as cholelithiasis, is a prevalent medical condition that has substantial global implications. Due to its numerous benefits, such as cheaper costs and shorter hospital stays, laparoscopic cholecystectomy (LC) has replaced open surgery as the most often performed surgical method for treating a range of biliary problems in wealthy countries. Any medical procedure's long-term patient well-being must be assessed, starting with the quality of life (QoL), patient satisfaction, and postoperative healing.

Aim: The current study aims to evaluate patient satisfaction and QoL after undergoing LC in Al-Qunfudhah Governorate.

Methods: A cross-sectional study was conducted targeting all patients who underwent LC in Al-Qunfudhah Governorate during the period from January to March 2024. The online survey was initiated by the study researchers after an intensive literature review and experts' consultation. The validated questionnaire was uploaded online using Google Forms and distributed electronically via social media apps.

Results: The current study included records of 200 participants where the highest proportion falls within the 26 to 35 age group, comprising 57 individuals (28.5%). In terms of gender, males represent the majority, with 109 participants (54.5%). A total of 122 individuals (61.0%) reported being satisfied with their procedures. Conversely, 18 patients (9.0%) expressed dissatisfaction. The majority of participants under investigation expressed satisfaction with their overall QoL after undergoing LC, with 84 patients (42.0%) reporting satisfaction and 67 patients (33.5%) reporting being very satisfied. Additionally, only a small proportion of participants expressed dissatisfaction or very dissatisfaction

Conclusion: In summary, the current study demonstrated high satisfaction with the LC treatment and highly reported QoL, which were mostly attributable to a number of factors such as the staff's cooperation, the lack of severe problems, and the sufficiency of the pre-surgery information supplied.

## Introduction

Cholecystitis is typified by inflammation of the gallbladder, a little organ that sits under the liver [1,2]. The most common cause is gallstones, which obstruct bile flow and cause inflammation and swelling of the gallbladder walls [1,3]. Severe stomach discomfort, tenderness in the upper right abdomen, nausea, vomiting, and fever are some of the symptoms brought on by this inflammation [4,5]. Diagnosing cholecystitis through physical examination, imaging studies, and blood work requires immediate medical intervention. Often, treatment entails removing the gallbladder by open or laparoscopic surgery [6,7].

A less invasive surgical procedure called laparoscopic cholecystectomy (LC) is used to remove the gallbladder [8,9]. For disorders of the gallbladder, including gallstones and several gallbladder illnesses, it has emerged as the gold standard of care. Compared to open surgery, which is recognized for its advantages in shortened hospital stays, quicker recovery periods, and less discomfort following surgery, this approach has advantages [10,11]. Even though LC is safe and effective, it is still critical to assess patient satisfaction and quality of life (QoL) after the treatment.

A key sign of any surgical intervention's success is patient satisfaction. Gaining an understanding of how patients view their post-LC experience can help drive future changes in patient care and offer important insights into the procedure's efficacy [12]. Patient satisfaction may be impacted by a number of factors, such as pain control, surgical site healing, cosmetic results, and the overall perceived benefits of the procedure. There is a need for a study evaluating satisfaction and QoL after undergoing LC in patients in Al-Qunfudhah Governorate, as there is limited research in this field. The objective of this study is to measure the level of patient satisfaction and QoL after undergoing LC in the Al-Qunfudhah Governorate by gathering the number of patients who had undergone LC.

## Materials and methods

A cross-sectional study was conducted targeting all patients who underwent LC in Al-Qunfudhah Governorate during the period from January to March 2024. Based on records, the sample size included 200 eligible patients from the Al-Qunfudhah Governorate of Saudi Arabia who had undergone the procedure during the study period. 

Data were collected using a self-administered online questionnaire, which will be followed by approval to ensure data confidentiality. The online survey was initiated by the study researchers after an intensive literature review and experts' consultation. The validated questionnaire was uploaded online using Google Forms and distributed electronically via social media apps. The survey will consist of multiple-choice questions. The questionnaire of this study included four sections. The first section covered the participants' demographic characteristics. The respondents were asked general questions about their LC in the second section. Thirdly, the questions were about satisfaction level, and finally, the last section assessed the improvement of the QoL post-procedure. Ethical approval was obtained from the Biomedical Research Ethics Committee of Umm Al-Qura University, Al-Qunfudhah, Saudi Arabia (approval number: HAPO-02-K-012-2024-01-1969).

Statistical analysis

The statistical analysis was conducted using RStudio software, version 4.3.1 (Posit PBC, Boston, MA). Descriptive statistics were used to summarize the variables, whereas categorical variables were presented as frequencies and percentages. To assess the factors associated with participants' satisfaction with LC and the perceptions regarding the QoL after the procedure, chi-squared tests or Fisher's exact tests were performed, as appropriate. Furthermore, binary logistic regression analysis was conducted to identify predictors of participants' satisfaction with their QoL. The significantly associated variables in the inferential analysis testing were considered independent variables. Odds ratios (ORs) along with 95% confidence intervals (CIs) were calculated to estimate the strength and direction of associations. A p-value of less than 0.05 was considered statistically significant.

## Results

Demographic characteristics

The current study included records of 200 participants who had undergone LC procedures. The age distribution shows that the highest proportion falls within the 26 to 35 age group, comprising 57 individuals (28.5%). In terms of gender, males represent the majority, with 109 participants (54.5%). Saudi nationals make up the largest portion of the sample, accounting for 173 individuals (86.5%). Educationally, individuals with a bachelor's degree constitute the largest group, totaling 65 participants (32.5%). When considering employment status, the employed category is predominant, with 91 participants (45.5%, Table 1).

**Table 1:** Demographic characteristics The values are given as n (%) Satisfaction with LC procedures and the associated factors

Characteristic	N=200
Age (years)	
12 to 17	10 (5.0%)
18 to 25	29 (14.5%)
26 to 35	57 (28.5%)
36 to 45	59 (29.5%)
46 to 60	33 (16.5%)
60 or more	12 (6.0%)
Gender	
Male	109 (54.5%)
Female	91 (45.5%)
Nationality	
Saudi	173 (86.5%)
Non-Saudi	27 (13.5%)
Educational level	
High school or less	54 (27.0%)
Diploma	50 (25.0%)
Bachelor's	65 (32.5%)
Master's	26 (13.0%)
PhD	5 (2.5%)
Employment status	
Student	28 (14.0%)
Employed	91 (45.5%)
Unemployed	63 (31.5%)
Retired	18 (9.0%)

**Figure 1:**
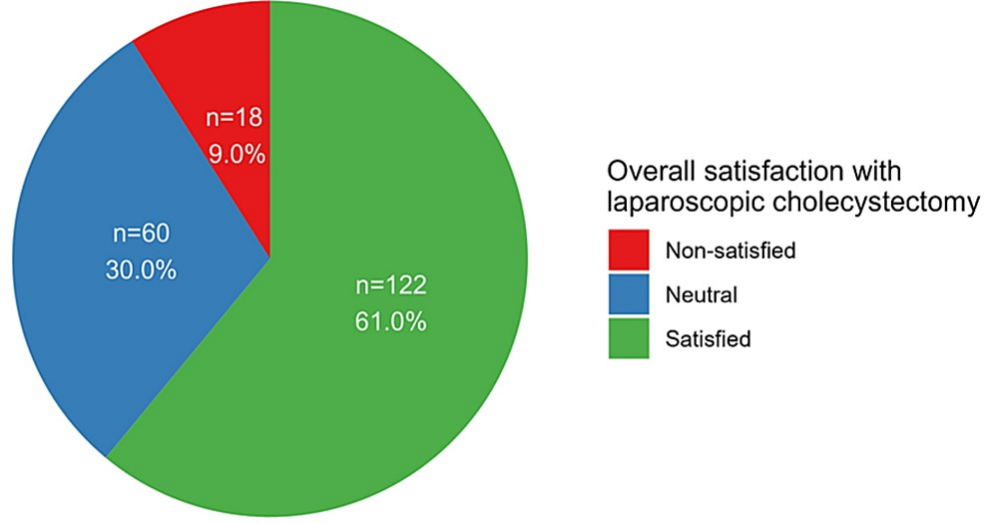
Overall satisfaction with laparoscopic cholecystectomy

In general, the majority of patients, comprising 122 individuals (61.0%), reported being satisfied with their procedures. Conversely, 18 patients (9.0%) expressed dissatisfaction, while 60 individuals (30.0%) provided neutral responses (Figure 1). Table 2 presents factors associated with participants' satisfaction following LC, where a marked contrast was evident between Saudi and non-Saudi participants, with a notably higher proportion of Saudi patients reporting satisfaction (115 patients, 66.5%) compared to non-Saudi patients (seven patients, 25.9%, p < 0.001).

**Table 2:** Factors associated with participants’ satisfaction with laparoscopic cholecystectomy The values are given as n (%) Fisher's exact test; Pearson's Chi-squared test

Characteristic	Satisfied with laparoscopic cholecystectomy		P-value
	No/Neutral, N=78	Yes, N=122	
Age (years)			0.323
12 to 17	5 (50.0%)	5 (50.0%)	
18 to 25	15 (51.7%)	14 (48.3%)	
26 to 35	20 (35.1%)	37 (64.9%)	
36 to 45	20 (33.9%)	39 (66.1%)	
46 to 60	11 (33.3%)	22 (66.7%)	
60 or more	7 (58.3%)	5 (41.7%)	
Gender			0.882
Male	42 (38.5%)	67 (61.5%)	
Female	36 (39.6%)	55 (60.4%)	
Nationality			<0.001
Saudi	58 (33.5%)	115 (66.5%)	
Non-Saudi	20 (74.1%)	7 (25.9%)	
Educational level			0.364
High school or less	19 (35.2%)	35 (64.8%)	
Diploma	19 (38.0%)	31 (62.0%)	
Bachelor's	24 (36.9%)	41 (63.1%)	
Master's	12 (46.2%)	14 (53.8%)	
PhD	4 (80.0%)	1 (20.0%)	
Employment status			0.187
Student	13 (46.4%)	15 (53.6%)	
Employed	36 (39.6%)	55 (60.4%)	
Unemployed	19 (30.2%)	44 (69.8%)	
Retired	10 (55.6%)	8 (44.4%)	

Participants’ responses regarding their general information regarding LC

Among the participants, the majority reported experiencing no complications or adverse effects following LC (143 patients, 71.5%). Additionally, most respondents rated their improvement in QoL since the procedure as either moderate (83 patients, 41.5%) or significant (63 patients, 31.5%). In terms of postoperative pain compared to expectations, the highest proportion indicated experiencing pain as expected (63 patients, 31.5%). Regarding satisfaction with postoperative follow-up care provided by healthcare professionals, a considerable number expressed either satisfaction (86 patients, 43.0%) or very satisfaction (60 patients, 30.0%). Furthermore, the majority reported no recurrence of gallstone-related symptoms after the procedure (145 patients, 72.5%). The majority of participants (132 patients, 66.0%) reported not having any pre-existing conditions that affected their recovery after LC. Concerning the level of information provided about the potential risks and benefits of LC before the procedure, the most common response was being moderately informed (68 patients, 34.0%, Table 3).

**Table 3:** Participants’ responses regarding their general information regarding laparoscopic cholecystectomy The values are given as n (%)

Characteristic	N=200
Experienced any complications or adverse effects following the laparoscopic cholecystectomy procedure	
No	143 (71.5%)
Yes	57 (28.5%)
How would you rate the improvement in your quality of life since undergoing laparoscopic cholecystectomy?	
No change	18 (9.0%)
Slight improvement	36 (18.0%)
Moderate improvement	83 (41.5%)
Significant improvement	63 (31.5%)
Compared to your expectations, how would you rate the postoperative pain after laparoscopic cholecystectomy?	
Much less than expected	12 (6.0%)
Less than expected	31 (15.5%)
As expected	63 (31.5%)
More than expected	61 (30.5%)
Much more than expected	33 (16.5%)
Experienced any limitations or difficulties in performing daily activities after laparoscopic cholecystectomy	
No	135 (67.5%)
Yes	65 (32.5%)
How satisfied are you with the postoperative follow-up care provided by healthcare professionals?	
Very dissatisfied	3 (1.5%)
Dissatisfied	13 (6.5%)
Neutral	38 (19.0%)
Satisfied	86 (43.0%)
Very Satisfied	60 (30.0%)
Have you experienced any recurrence of gallstone-related symptoms after laparoscopic cholecystectomy?	
No	145 (72.5%)
Yes	55 (27.5%)
Did you have any pre-existing conditions that affected your recovery after laparoscopic cholecystectomy?	
No	132 (66.0%)
Yes	68 (34.0%)
How well were you informed about the potential risks and benefits of laparoscopic cholecystectomy before the procedure?	
Not informed at all	14 (7.0%)
Poorly informed	27 (13.5%)
Moderately informed	68 (34.0%)
Well informed	48 (24.0%)
Very informed	43 (21.5%)

Participants’ responses regarding LC healthcare team provider

Among the sample under study, the majority rated the communication with the surgical team before the procedure as either good (84 patients, 42.0%) or excellent (65 patients, 32.5%). Furthermore, most respondents reported being given adequate information regarding the preoperative preparations for LC (131 patients, 65.5%). Concerning the responsiveness of healthcare professionals to concerns and questions before the procedure, a notable proportion indicated either responsiveness (93 patients, 46.5%) or very responsiveness (53 patients, 26.5%). Regarding the clarity of instructions provided for postoperative care, responses varied with a significant portion finding instructions either clear (74 patients, 37.0%) or very clear (52 patients, 26.0%). The majority reported feeling supported by the healthcare team during the recovery period after LC (129 patients, 64.5%). Additionally, most participants expressed satisfaction with the information provided about potential complications and their management after the procedure (82 patients, 41.0%). When rating the overall professionalism and competence of the healthcare team involved in their LC, a substantial majority rated it as either good (78 patients, 39.0%) or excellent (84 patients, 42.0%). Lastly, most respondents reported that the healthcare team addressed their pain management needs effectively after LC (147 patients, 73.5%, Table 4).

**Table 4:** Participants’ satisfaction regarding laparoscopic cholecystectomy surgical team The values are given as n (%)

Characteristic	N=200	
How would you rate the communication between you and the surgical team before the procedure?		
Poor	11 (5.5%)	
Fair	40 (20.0%)	
Good	84 (42.0%)	
Excellent	65 (32.5%)	
Were you given adequate information regarding the preoperative preparations for laparoscopic cholecystectomy?		
No	69 (34.5%)	
Yes	131 (65.5%)	
How would you rate the responsiveness of healthcare professionals to your concerns and questions before the procedure?		
Not responsive at all	4 (2.0%)	
Not very responsive	14 (7.0%)	
Neutral	36 (18.0%)	
Responsive	93 (46.5%)	
Very responsive	53 (26.5%)	
How would you rate the clarity of instructions provided for postoperative care?		
Very unclear	6 (3.0%)	
Unclear	12 (6.0%)	
Neutral	56 (28.0%)	
Clear	74 (37.0%)	
Very clear	52 (26.0%)	
Did you feel supported by the healthcare team during the recovery period after laparoscopic cholecystectomy?		
No	71 (35.5%)	
Yes	129 (64.5%)	
How satisfied were you with the information provided about potential complications and their management after the procedure?		
Very dissatisfied	9 (4.5%)	
Dissatisfied	19 (9.5%)	
Neutral	48 (24.0%)	
Satisfied	82 (41.0%)	
Very Satisfied	42 (21.0%)	
How would you rate the overall professionalism and competence of the healthcare team involved in your laparoscopic cholecystectomy?		
Poor	10 (5.0%)	
Fair	28 (14.0%)	
Good	78 (39.0%)	
Excellent	84 (42.0%)	
Did the healthcare team address your pain management needs effectively after laparoscopic cholecystectomy?		
No	53 (26.5%)	
Yes	147 (73.5%)	

Participants’ responses regarding their self-reported QoL after LC

A considerable proportion of patients rated their overall physical well-being after LC as either good (84 patients, 42.0%) or excellent (67 patients, 33.5%). Additionally, a significant proportion reported experiencing improvements in digestion and appetite after the procedure (128 patients, 64.0%). Regarding satisfaction with the ability to engage in physical activities, most respondents expressed either satisfaction (82 patients, 41.0%) or very satisfaction (55 patients, 27.5%). In terms of mental and emotional well-being, a substantial majority rated it as either good (80 patients, 40.0%) or excellent (87 patients, 43.5%). Furthermore, the majority reported no changes in their sleep patterns or quality of sleep since undergoing LC (107 patients, 53.5%). When assessing the impact on the ability to perform daily activities and tasks, a significant proportion reported moderate improvement (89 patients, 44.5%). Lastly, most respondents rated their social interactions and relationships after LC as either good (72 patients, 36.0%) or excellent (93 patients, 46.5%, Table 5).

**Table 5:** Participants’ responses regarding their self-reported quality of life after laparoscopic cholecystectomy The values are given as n (%)

Characteristic	N=200
How would you rate your overall physical well-being after laparoscopic cholecystectomy?	
Poor	8 (4.0%)
Fair	41 (20.5%)
Good	84 (42.0%)
Excellent	67 (33.5%)
Did you experience any improvements in your digestion and appetite after laparoscopic cholecystectomy?	
No	72 (36.0%)
Yes	128 (64.0%)
How satisfied are you with your ability to engage in physical activities after laparoscopic cholecystectomy?	
Very dissatisfied	5 (2.5%)
Dissatisfied	13 (6.5%)
Neutral	45 (22.5%)
Satisfied	82 (41.0%)
Very Satisfied	55 (27.5%)
How would you rate your mental and emotional well-being after laparoscopic cholecystectomy?	
Poor	9 (4.5%)
Fair	24 (12.0%)
Good	80 (40.0%)
Excellent	87 (43.5%)
Have you noticed any changes in your sleep patterns or quality of sleep since undergoing laparoscopic cholecystectomy?	
No	107 (53.5%)
Yes	93 (46.5%)
Did laparoscopic cholecystectomy improve your ability to perform daily activities and tasks?	
No change	22 (11.0%)
Yes, slightly	34 (17.0%)
Yes, moderately	89 (44.5%)
Yes, significantly	55 (27.5%)
How would you rate your social interactions and relationships after laparoscopic cholecystectomy?	
Poor	9 (4.5%)
Fair	26 (13.0%)
Good	72 (36.0%)
Excellent	93 (46.5%)
Have you experienced any negative effects on your body image or self-esteem following laparoscopic cholecystectomy?	
No	138 (69.0%)
Yes	62 (31.0%)

**Figure 2:**
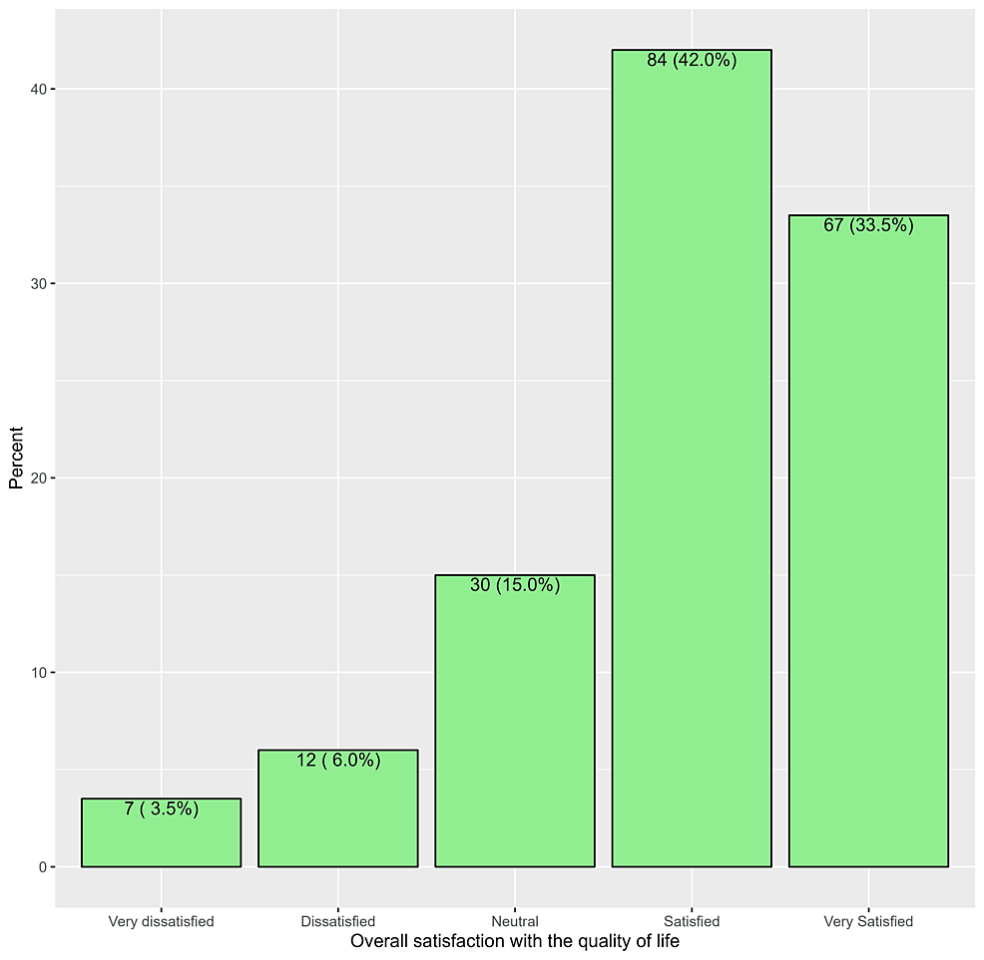
Overall satisfaction with the quality of life

Importantly, the majority of participants under investigation expressed satisfaction with their overall QoL after undergoing LC, with 84 patients (42.0%) reporting satisfaction and 67 patients (33.5%) reporting being very satisfied. Additionally, only a small proportion of participants expressed dissatisfaction or very dissatisfaction, with 12 patients (6.0%) reporting dissatisfaction and seven patients (3.5%) reporting being very dissatisfied. Furthermore, a moderate number of respondents indicated a neutral stance, with 30 patients (15.0%) expressing neutrality towards their QoL post-surgery (Figure 2).

Table 6 presents factors associated with participants' satisfaction with the QoL after LC. Notably, there was a significant difference in the perceived QoL in terms of education levels, with higher proportions of improved QoL among patients with a high school degree or less (47 patients, 90.7%) and Master’s degree (21 patients, 80.8%) compared to those with the following degrees: diploma (37 patients, 74.0%), Bachelor’s (41 patients, 63.1%) and PhD (three patients, 60.0%, p = 0.005). Moreover, a substantial difference in satisfaction was observed concerning participants' satisfaction with the LC procedure itself, with a significantly higher proportion of satisfied individuals reporting satisfaction with their QoL (104 patients, 85.2% vs 47 patients, 60.3% among those with no or neutral satisfaction, p < 0.001). Other variables, including age, gender, nationality, and employment status, did not show significant associations with satisfaction levels (p > 0.05, Table 6).

**Table 6:** Factors associated with participants’ satisfaction with the quality of life after laparoscopic cholecystectomy n (%) Fisher's exact test; Pearson's Chi-squared test

Characteristic	Satisfied with the quality of life		P-value
	No/Neutral, N=49	Yes, N=151	
Age (years)			0.729
12 to 17	2 (20.0%)	8 (80.0%)	
18 to 25	8 (27.6%)	21 (72.4%)	
26 to 35	18 (31.6%)	39 (68.4%)	
36 to 45	13 (22.0%)	46 (78.0%)	
46 to 60	6 (18.2%)	27 (81.8%)	
60 or more	2 (16.7%)	10 (83.3%)	
Gender			0.277
Male	30 (27.5%)	79 (72.5%)	
Female	19 (20.9%)	72 (79.1%)	
Nationality			0.505
Saudi	41 (23.7%)	132 (76.3%)	
Non-Saudi	8 (29.6%)	19 (70.4%)	
Educational level			0.005
High school or less	5 (9.3%)	49 (90.7%)	
Diploma	13 (26.0%)	37 (74.0%)	
Bachelor's	24 (36.9%)	41 (63.1%)	
Master's	5 (19.2%)	21 (80.8%)	
PhD	2 (40.0%)	3 (60.0%)	
Employment status			0.486
Student	6 (21.4%)	22 (78.6%)	
Employed	26 (28.6%)	65 (71.4%)	
Unemployed	15 (23.8%)	48 (76.2%)	
Retired	2 (11.1%)	16 (88.9%)	
Satisfied with the laparoscopic cholecystectomy procedure			<0.001
No/Neutral	31 (39.7%)	47 (60.3%)	
Yes	18 (14.8%)	104 (85.2%)	

In the regression model investigating the predictors of favorable perceptions of the QoL postoperatively, results showed that participants with a diploma level of education had lower odds of being satisfied with their QoL after the surgery compared to those with a high school education or less (OR=0.28, 95% CI: 0.08, 0.84, p = 0.029). Similarly, individuals with a bachelor's degree had significantly lower odds of satisfaction (OR=0.15, 95% CI: 0.05, 0.43, p < 0.001). Regarding satisfaction with the LC procedure itself, those who reported being satisfied had substantially higher odds of higher perceptions regarding their QoL (OR=4.23, 95% CI: 2.09, 8.87, p < 0.001, Table 7).

**Table 7:** Predictors of participants’ satisfaction with the quality of life after laparoscopic cholecystectomy OR = Odds Ratio, CI = Confidence Interval

Characteristic	OR	95% CI	P-value
Educational level			
High school or less	Reference	Reference	
Diploma	0.28	0.08, 0.84	0.029
Bachelor's	0.15	0.05, 0.43	<0.001
Master's	0.48	0.12, 1.97	0.299
PhD	0.25	0.03, 2.34	0.192
Satisfied with the laparoscopic cholecystectomy procedure			
No/Neutral	Reference	Reference	
Yes	4.23	2.09, 8.87	<0.001

## Discussion

The current study aimed to assess patient satisfaction and QoL after undergoing LC in Al-Qunfudhah Governorate, Saudi Arabia. Since the early 1990s, LC, a less invasive procedure for removing a diseased gallbladder, has become more popular than the open approach because of developments in medical technology [13]. This treatment is currently the standard approach for a range of illnesses, ranging from symptomatic cholelithiasis and chronic cholecystitis to more severe problems such as gallstone pancreatitis [14]. LC is extensively utilized in high-income nations due to its numerous benefits [15], including reduced hospital stays, quicker recovery periods, and decreased expenses [16]. The safety of this product is remarkable, with an impressively low mortality rate ranging from 0.22% to 0.4% [17].

The current study showed that most of the study patients undergone LC were at their middle ages, males, with high education. Regarding post-LC satisfaction rate, the study results revealed that about two-thirds of them (61%) were satisfied with LC mainly Saudi patients but no gender or age differences regarding satisfaction were reported. This high satisfaction is mostly associated with that the vast majority of the cases had no complications after LC with an expected pain threshold. A much higher satisfaction was reported in Rwanda where overall satisfaction exceeded 95% and the vast majority of patients consider laparoscopic surgery as the best surgical approach [18]. Another study by Saber and Hokkam [19] revealed that early LC resulted in a significant reduction in LOS an acceptable rate of operative complications and conversion rates and overall patient satisfaction between 75% and 93%. In Saudi Arabia, Aleid et al. [20] found that patients showed satisfaction with LC; however, complications were reported in a significant number of cases. However, a minority of participants were dissatisfied with LC. The authors also found no significant gender differences in post-surgery satisfaction which is consistence with the current study findings. Another study by Terro et al. [21] found that most of the patients were satisfied with postoperative pain management and their cosmetic appearance. Also, numerous studies have demonstrated that, despite an overall serious complication rate of 5%, which is still higher than that of an open cholecystectomy, LC has become more widely accepted with a higher satisfaction rate which may exceed 90% [22-25]. Other studies revealed high satisfaction due to better cosmetic outcomes in LC compared to surgical procedures [26,27].

As for the post-LC QoL, the current study showed that about three-fourths (75.5%) of the study patients were satisfied with the QoL and this explains the reported high satisfaction with the procedure. Low education with satisfaction about the procedure itself was significantly associated with perceived high QoL. This can be due to the fact that low-educated persons have a lower threshold for their expectations regarding any undergone procedure compared to highly educated persons who mostly have high expectations [28,29]. Also, improved appetite, physical activity, and sleep patterns were among the factors that improved their QoL. Similarly, in Saudi Arabia, Aleid et al. [20] documented that the majority of respondents said their QoL had moderately improved after LC. Demographic characteristics such as gender, age, and employment position had significant effects on satisfaction and QoL. In concordance with the current study findings, Mattila et al. [16] found that after LC, all patient groups reported notable reductions in physical discomfort and increased energy. Orthopedic and inguinal hernia patients experienced a considerable improvement in their physical performance. Postoperative physical health scores in the orthopedic groups did, however, remain comparatively lower than the reference values for the general population. Similar findings were also reported by Lamberts et al. [30]. Another study conducted by Bohara et al. [31] revealed a significant increase in QoL after LC in symptomatic patients. Likewise, Gach et al. [32] found that 84.9% of the symptomatic group experienced an improvement in their health condition and 5.7% remained unchanged. These reported high QoL afire LC were also approved by many other researchers in the literature indicating the procedure's high effectiveness and acceptance [33-35].

With regard to participants' perception regarding the procedure and healthcare staff, high satisfaction with the staff's role in providing information before surgery, response to explanations, good communication, and high support. This also explains their high satisfaction and post-LC-related QoL. The study may have a limited sample size due to various factors such as resource constraints, time limitations, or a small population in Al-Qunfudhah Governorate. A small sample size may limit the generalizability of the findings to a larger population. Also, the study may have a limited follow-up duration, which could impact the ability to capture long-term outcomes and changes in patient satisfaction and QoL. Longer follow-up periods provide a more comprehensive understanding of the postoperative effects of LC.

## Conclusions

This study showed a high level of satisfaction with the LC procedure with its good impact on patient QoL due to its lack of complications, cooperation of the staff, and adequacy of pre-surgery provided information. Interestingly, our work revealed that age and gender may not have a substantial impact on postoperative QoL evaluations in the Saudi setting. In the future, it is necessary to fully assess LC versus alternative therapies and dig deeper into patient experiences.
